# Autograft Polarity on Functional Outcomes Following Experimental Peripheral Nerve Repair Surgery: A Systematic Review and Meta-Analysis

**DOI:** 10.3390/jcm14248885

**Published:** 2025-12-16

**Authors:** Hye Ok Kim, Yeon Ju Oh, Sung Soo Kim, Youn-Jung Kim, Jin Woo Choi, Jae Min Lee, Seung Geun Yeo

**Affiliations:** 1 Department of Medicine, College of Medicine, Kyung Hee University, Seoul 02447, Republic of Korea; hyeokkim@khu.ac.kr (H.O.K.); 5duswn1203@khu.ac.kr (Y.J.O.); 2 Clinical Research Institute, Kyung Hee University Medical Center, Seoul 02447, Republic of Korea; 3 Department of Biochemistry and Molecular Biology, College of Medicine, Kyung Hee University, Seoul 02447, Republic of Korea; sgskim@khu.ac.kr; 4 College of Nursing Science, Kyung Hee University, Seoul 02447, Republic of Korea; yj129@khu.ac.kr (Y.-J.K.); jmlee3042@khu.ac.kr (J.M.L.); 5 Department of Pharmacology, College of Pharmacy, Kyung Hee University, Seoul 02447, Republic of Korea; jinwoo.ch@khu.ac.kr; 6 Department of Otorhinolaryngology Head & Neck Surgery, College of Medicine, Kyung Hee University Medical Center, Seoul 02447, Republic of Korea; 7 Department of Precision Medicine, Graduate School, Kyung Hee University, Seoul 02447, Republic of Korea; 8 Department of Convergence Medicine, College of Medicine, Kyung Hee University, Seoul 02447, Republic of Korea

**Keywords:** graft orientation, nerve regeneration, meta-analysis, animal model, peripheral nerve injury, nerve autograft, nerve conduction velocity

## Abstract

**Background:** Proximal–distal orientation of nerve autografts (graft polarity) is an important consideration in peripheral nerve repair, but the literature is inconsistent on whether reversing graft polarity improves regeneration. With these disparities, we aimed to systematically review the effect of forward (normal proximal-to-distal) vs. reversed graft orientation on peripheral nerve regeneration or functional outcomes. **Methods:** We conducted a comprehensive search of PubMed, Scopus, Cochrane Library, EMBASE, and Google Scholar (January 1978–August 2025) for studies comparing autograft polarity in nerve regeneration. Of 90 articles identified, 9 studies met inclusion criteria (comparative studies directly evaluating graft orientation). Data on nerve conduction and functional recovery outcomes were extracted, and random-effects meta-analyses (using Hedges’ g standardized mean differences) were performed to compare forward vs. reversed graft orientation, with heterogeneity assessed by I^2^ and τ^2^. **Results:** The nine included studies (all in animals) assessed histological, morphometric, electrophysiological (e.g., nerve conduction velocity, action potential amplitude), behavioral and functional outcomes. Three of the nine studies reported a significant outcome difference favoring one graft orientation, whereas six studies found no significant difference. Meta-analysis of five studies reporting nerve conduction velocity found no overall difference between forward and reversed orientation (Hedges’ g = −0.57, 95% confidence interval −1.52 to 0.37; *p* = 0.23; I^2^ = 82.96%, τ^2^ = 0.935). **Conclusions:** This meta-analysis provides the first quantitative synthesis of experimental evidence assessing the impact of nerve autograft orientation, revealing no consistent advantage of forward or reversed polarity on regeneration outcomes. Although based on limited and heterogeneous animal data, the findings clarify existing trends and highlight areas for further experimental and clinical research.

## 1. Introduction

Peripheral nerves are highly susceptible to injury and can be easily harmed by acute compression or trauma. In particular, complete nerve transection (axotomy) represents a severe form of damage, and achieving even partial recovery in such cases remains challenging [1]. When conservative management proves inadequate, surgical procedures such as nerve reconstruction are often required [2]. Although surgical outcomes vary with the severity of the injury, achieving complete recovery is particularly challenging in cases of total nerve transection [3]. Furthermore, in cases of complete facial nerve transection, even the most advanced interventions rarely achieve functional recovery beyond a moderate level (House–Brackmann Grade III), reflecting the limitations of current treatment strategies for such severe nerve injuries [4]. Peripheral nerve injuries are typically managed using nerve suturing or grafting techniques [5]. In cases where direct suturing cannot be performed, nerve grafts serve as conduits for axonal regeneration and facilitate the restoration of neural function. Achieving successful nerve reconstruction is critical for functional recovery and can greatly enhance the patient’s quality of life [6].

In autologous nerve grafting, orientation strongly influences nerve regeneration [7]. The forward-suture technique (aligning the distal and proximal ends in the same direction) facilitates proper signal transmission and often yields more effective functional recovery [8], whereas the reverse-suture technique (connecting the ends in opposite directions) inverts the nerve fibers [9]. Although this inverted configuration does not inherently promote nerve regeneration, the continuous structure of the graft still provides a conduit that allows some axonal growth and eventual functional recovery [10].

According to previous reports, many surgeons reverse the polarity of the autograft during autogenous nerve grafting, with the intent of improving nerve regeneration by mitigating the potential misrouting effects of arborization. However, the optimal orientation of an autogenous nerve graft remains inconsistent [11].

Stromberg et al. [12] compared nerve graft polarity in rat using the sciatic nerve. They determined that the function of the nerve graft was independent of its polarity. In the study of Nakatsuka et al. [13], which assessed the effect of cable nerve graft polarity, there was no significant difference in either motor conduction velocity or muscle weight as a functional outcome of nerve graft orientation. However, Ansselin and Davey [14,15] concluded that regeneration of axons to their peripheral targets is facilitated by reversing the graft orientation. This was disputed by Millesi [16], who found that nerve graft reversal did not enhance regeneration. In a systematic review of the effect of autograft polarity on functional outcomes following peripheral nerve repair surgery, it was concluded that there were insufficient data suggesting that the polarity of an autologous nerve graft impacts on nerve regeneration and functional outcome. Furthermore, only six studies were included in that review, and most of them were conducted more than 20 years ago [17]. Then, it is necessary to comprehensive analysis that incorporates not only previous findings but also recent research trends.

This study aimed to conduct a meta-analysis to evaluate the impact of forward versus reverse suture techniques—where the former maintains the native orientation of nerve fibers and the latter inverts them—on nerve regeneration following axotomy. Therefore, we conducted a meta-analysis using data from several assessment modalities measuring nerve regeneration in animal models.

## 2. Materials and Methods

### 2.1. Search Strategy

**Figure 1:**
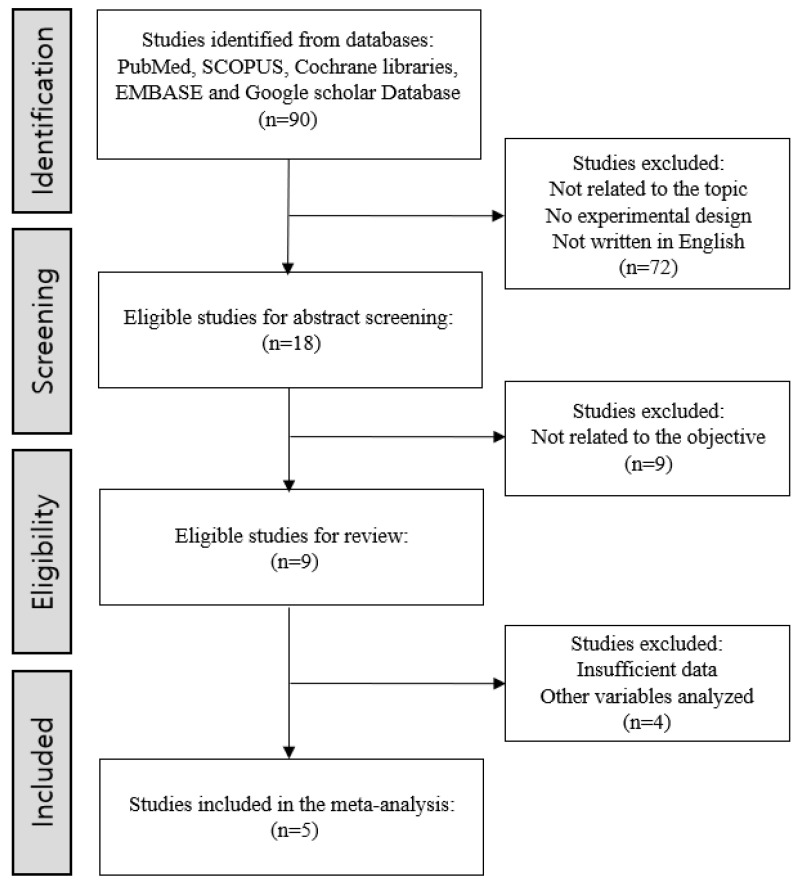
PRISMA flowchart for study selection.

A systematic review and meta-analysis were conducted following PRISMA guidelines, and the protocol was registered on OSF (accessed on 18 November 2025; https://osf.io/a6h8p/overview; Supplementary Table S1). We searched multiple databases (PubMed/MEDLINE, Scopus, Cochrane CENTRAL, EMBASE, and Google Scholar) from database inception (January 1978) through August 2025 to identify all relevant studies. The search strategy was based on the key concepts of the review (e.g., “nerve axotomy” and “nerve graft”) and included synonyms and related terms. These terms were combined using Boolean operators; for example, the strategy included (‘nerve axotomy’ AND ‘suture’) OR ‘nerve graft orientation’ OR ‘nerve graft polarity’ OR ‘nerve graft reversal’ to capture variations in terminology. No date restrictions were applied (beyond the database coverage), but the search was limited to English-language articles in accordance with our eligibility criteria. After removing duplicates, the remaining records were screened by title, abstract, and full text to exclude non-experimental or irrelevant studies. In total, 9 studies met the inclusion criteria for the systematic review and 5 were included in the meta-analysis. The process of study selection is summarized in the PRISMA flow diagram (Figure 1).

### 2.2. Article Eligibility

The inclusion criteria for this study were articles that were (1) peer-reviewed comparative studies, such as randomized controlled trials and observational studies; (2) direct comparisons of nerve graft orientation: normal proximal-distal versus reversed orientation; (3) evaluations of the effect of orientation with functional, histologic, or clinic metrics. Three reviewers (S.Y., H.K., and Y.O.) independently scanned the retrieved abstracts and evaluated the potential relevance. The reviewers were able to achieve consensus in article relevancy. An overview of the methods and findings for the studies are reported in Table 1 [6,11,12,13,14,15,18,19,20], respectively.

### 2.3. Data Extraction

In this review, the studies included evaluated the effect of nerve graft orientation in animal models. We did not identify any human studies that met our inclusion criteria. These studies included the following evaluations: gross measurements, histopathology and morphometry, electrophysiological recovery, behavioral/functional assessment. The following data were extracted from each primary article. Two independent authors (H.K., Y.O.) extracted the following variables: author, year and journal, title, model, sample size, nerve site, assessment modality (axon diameter, thickness, cross-sectional area, conduction velocity, action potential (amplitude), neuron counts, axon diameter, weight of muscle), and conclusions. A third author (S.Y.) double-checked these data. The main outcome analyzed was the effect of autograft polarity on nerve regeneration. Data were collected based on the measured assessment results to determine the effects of two possible scenarios: forward and reverse suture on nerve regeneration after axotomy. All data are summarized in Table 1.

### 2.4. Data Analysis

**Figure 2:**
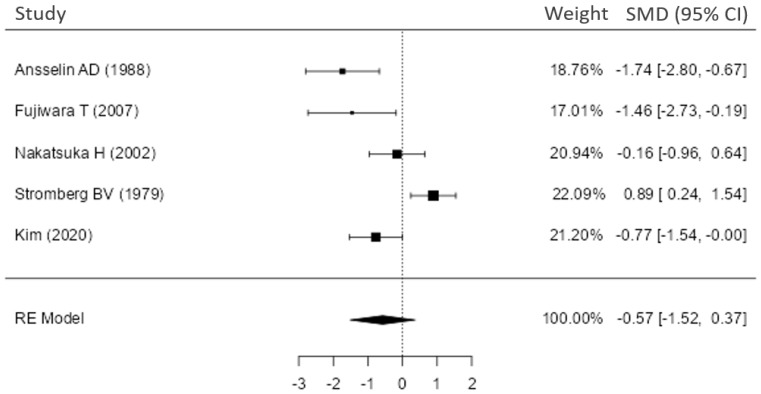
Meta-analysis of nerve conduction velocity (NCV) in forward and reversed nerve graft orientation Forest plot of meta-analysis (Conduction Velocity). Each study’s effect size (Hedges’ g) is shown as a black square, with horizontal lines indicating the 95% confidence interval. The vertical dashed grey line at 0 represents no effect (no difference in conduction velocity between forward and reverse polarity groups). The size of each square is proportional to the study’s weight in the meta-analysis. The overall combined effect (random-effects model) is shown as a black diamond, with its width spanning the 95% confidence interval for the pooled Hedges’ g. Test for overall effect: Z = −1.19 (*p* = 0.23); Heterogeneity: Q = 24.6, df = 4.0 (*p* < 0.001), τ^2^ = 0.935, I^2^ = 82.96%, [11,12,13,14,18].

The outcome measure used in this analysis was the standardized mean difference (SMD), and a random-effects model was applied to the data. Heterogeneity (tau^2^) was estimated using the restricted maximum likelihood (REML) method. Additionally, heterogeneity statistics including the Q-test and I^2^ value were reported and are presented in Figure 2. To assess the publication bias, we examined funnel plots and performed Egger’s regression and Begg’s rank correlation test (Figure S1). In tests, values of *p* < 0.05 were considered statistically significant. Microsoft Excel was used to prepare a database and for statistical analysis; second, Jamovi (version 2.6.44) software was used to conduct the meta-analysis.

## 3. Results

The PRISMA 2020 flow diagram (Figure 1) outlines the stages of identification, screening, and eligibility assessment used to select studies for inclusion in the review. An initial search of multiple databases identified 90 records; after removal of duplicates and screening for eligibility, nine studies met the inclusion criteria and were included in the final analysis. The characteristics and findings of these nine included animal studies (Table 1) are briefly summarized below, including details of their study designs, animal models (rat or rabbit), interventions, and outcome measures (electrophysiological, histological, and functional).

Nine animal studies comparing forward vs. reversed nerve autograft orientation in peripheral nerve repair are included in this review, comprising seven rat and two rabbit studies with sample sizes ranging from 12 to 62 animals each. Most studies employed a sciatic nerve transection and autograft repair model in rats, whereas one study examined facial nerve repair in rats and two utilized hindlimb nerve repair models in rabbits (common peroneal nerve and tibial nerve grafts). These studies evaluated a wide range of outcome measures, including electrophysiological indices (nerve conduction velocity and compound action potential amplitude), histomorphometric parameters (axon diameter, myelin thickness, axon counts or density, cross-sectional area), muscle re-innervation measures (muscle contraction force and muscle weight), and functional recovery outcomes (e.g., the sciatic functional index). Three studies found that reversed (antidromic) autograft orientation improve axonal regeneration or function, while the remaining six studies reported no significant difference between forward (orthodromic) and reversed graft orientations (Table 1 and Table 2).

Five studies (total n = 123 nerve repairs) were included in a random-effects meta-analysis of nerve conduction velocity comparing forward (orthodromic) vs. reversed (antidromic) graft orientations (Figure 2). The pooled standardized mean difference (Hedges’ g) was −0.57 (95% CI −1.52 to 0.37), indicating a moderate trend favoring the reversed orientation, but this overall effect was not statistically significant (Z = −1.19, *p* = 0.23). There was significant heterogeneity between study (Cochran’s Q = 24.6, df = 4, *p* < 0.001; I^2^ = 82.96%, τ^2^ = 0.935). Individually, two studies showed significantly higher NCV with reversed grafts, whereas one study found a significant advantage for forward grafts. The remaining two studies reported no significant difference in NCV between forward and reversed graft orientations.

## 4. Discussion

In this systematic analysis of nine animal studies, we found that the proximodistal orientation of a nerve autograft (forward/orthodromic vs. reversed/antidromic) has minimal influence on overall regeneration outcomes. As shown in Table 1, six of the nine studies reported no significant differences in nerve recovery between forward and reversed grafts, while three studies observed some improvement in regeneration with reversed graft orientation. Notably, our meta-analysis of nerve conduction velocity (NCV) (Figure 2) revealed no statistically significant overall effect of graft polarity. The pooled effect size showed a modest trend favoring reversed grafts (Hedges’ g ≈ –0.57), but the confidence interval crossed zero and the overall difference was not significant (Z = −1.19, *p* = 0.23). There was substantial inter-study heterogeneity (I^2^ ≈ 85%), reflecting variability in individual study results—some experiments favored reversed grafts, others showed no difference, and one even favored the forward orientation. Taken together, these findings indicate that, while reversed orientation may offer benefits under specific conditions, autograft polarity does not have a major impact on overall regeneration. In other words, simply flipping a nerve graft 180° is unlikely to drastically improve or impair the nerve’s ability to regenerate across the graft.

Our results are largely consistent with the majority of previously published animal studies on this topic. Early work by Sanders and Young (1943) in rabbits and by Stromberg et al. (1979) in rats found that nerve grafts achieved comparable re-innervation regardless of orientation, with no meaningful differences in histology or functional recovery between normally oriented and reversed grafts [12,21]. In addition, Sotereanos et al. (1992) reported that motor functional outcomes were equivalent in a rat sciatic nerve model, with no difference in Sciatic Functional Index between forward vs. reversed grafts (*p* > 0.1) [19]. More recent investigations have reinforced this lack of disparity. For example, Nakatsuka et al. (2002) found no significant differences between forward and reversed cable grafts in a rabbit peroneal nerve repair model—nerve conduction velocities, muscle re-innervation (muscle weights of target muscles), and axon counts were all statistically similar between the two graft orientations [13]. Similarly, Kim et al. (2020) likewise observed that autograft polarity did not affect electrophysiological outcomes (compound motor action potential amplitude or NCV) in a rabbit tibial-to-peroneal nerve graft model [11]. Consistent with these reports, a rodent study by Afshari A et al. (2018) concluded that reversal of nerve autograft polarity using advanced MRI tracking (diffusion tensor imaging) did not affect regenerative outcomes or functional recovery [22]. Lee et al. (2024) also reported that the direction of suturing did not significantly affect nerve regeneration or functional recovery, indicating that graft orientation may not be a determining factor in the nerve repair process [6]. Musa Ergin et al. (2025) also demonstrated that normal autograft orientation is superior in sensory recovery, while no significant differences are observed in motor function or histological results [20]. Overall, the prevailing evidence—including the present meta-analysis—indicates that graft orientation by itself is not a major determinant of nerve repair success.

It is worth noting, however, that a few studies in the literature did suggest benefits to using reversed grafts, which initially fueled debate on this issue. In a series of rat sciatic nerve experiments, Ansselin and Davey reported that reversed (antidromic) grafts resulted in superior histomorphometric and electrophysiological outcomes. Their 1988 study found that antidromically oriented grafts yielded a significantly larger distal nerve cross-sectional area compared to normally oriented grafts (*p* = 0.002) [14], indicating more robust tissue regrowth in the graft. In 1993, the same group showed that nerves repaired with reversed grafts had significantly higher NCV and greater distal axon counts than forward-oriented grafts (*p* < 0.05 and *p* < 0.01, respectively) [15]. Despite these advantages in nerve fiber regeneration, they noted that ultimate motor functional recovery (evaluated by the Sciatic Functional Index) remained similar between the forward and reversed groups (*p* > 0.05). Fujiwara et al. (2007) also reported improved electrophysiological metrics using a “reverse end-to-side” graft technique in rats: adding short reversed autograft in an end-to-side fashion appeared to increase NCV and compound action potential amplitude in the repaired nerve [18]. However, the statistical significance of these improvements was not clearly demonstrated, and this approach represents a specialized scenario (a so-called nerve “supercharging” technique) rather than a typical end-to-end graft repair. Apart from these studies, virtually all other animal experiments—spanning different species, nerve injury models, and outcome measures—have found no clear regenerative advantage to either normal or reversed graft polarity [17]. The current findings therefore reconcile well with the broader literature: they confirm that any enhancements observed with reversed grafts in certain contexts do not translate into a generalized rule for improved nerve repair, especially in terms of functional recovery.

While some studies found differences in reversed grafts, most did not. One biological factor explaining these findings is axonal branching within the graft. In a normally oriented autograft, these branch points face in the distal direction. As regenerating axons grow through the graft, they can encounter these branch openings, and some axonal sprouts may divert into the side branches (which typically lead to dead ends or non-functional pathways). In a reversed graft, however, the orientation of those branch stumps is flipped. Consequently, axons are less likely to enter extraneous branches in a reversed graft. According to Ansselin and Davey, the reversed grafts ended up with significantly more axons present in the distal portion of the graft than the forward-oriented grafts [15]. Our results suggest that using a reverse graft orientation in nerve transplantation may maximize axonal regeneration and prevent misconnection, especially when grafting highly branched nerves. Clinically, if the harvested nerve graft contains one or more significant branch points, intentionally reversing the graft orientation may reduce the likelihood of regenerative axons exiting these branches [17]. However, these findings also suggest that surgeons need not be overly concerned about maintaining the original proximal–distal orientation of the autograft during nerve grafting.

There are several important limitations. First, our conclusions are based on animal model data, which may not capture all variables relevant to human nerve injuries. Rats, mice, and rabbits heal faster and over shorter nerve distances than humans do, and their peripheral nerves differ in scale and possibly in regenerative capacity. Included studies were conducted in animals over a short period of time (approximately 6–12 weeks for rodent models, or up to 6 months for rabbit models). One should be cautious in directly applying quantitative effects (or lack thereof) from these animal studies to human patients. Further research will be needed for practical clinical application. Second, there was notable heterogeneity among the included studies, which complicates interpretation of the pooled results. The experiments varied in species (six studies in rodents vs. two in rabbits), in the nerve type and location (e.g., sciatic nerve vs. facial nerve vs. peroneal nerve), in the surgical technique, in graft length and the presence of branch points, and in the outcomes measured (ranging from axon counts and histomorphology to muscle force and behavioral indices). This heterogeneity was reflected in our meta-analysis by a high I^2^ value (approximately 85%), indicating substantial variability in the results of individual studies. It suggests that subtle effects of orientation might exist under specific conditions but were diluted in the aggregate data. For example, one study using a facial nerve repair model found no difference between forward and reversed grafts [6], whereas certain sciatic nerve studies did find differences—these context-specific outcomes are difficult to reconcile and contribute to the overall statistical heterogeneity. Furthermore, the funnel plot also showed asymmetry, suggesting potential publication bias. Egger’s regression test was statistically significant (*p* = 0.007), suggesting a small-study effect, whereas Begg’s test was not significant (*p* = 0.233). However, with only five studies included in the meta-analysis, the power of these tests to detect such bias is limited (Figure S1). In this context, the discrepancy between Egger’s and Begg’s test results likely reflects the high heterogeneity among the included studies; accordingly, these findings can only imply a possible publication bias rather than providing definitive evidence of one. Third, variations in surgical execution across studies may have influenced the outcomes. The experience of the surgeon performing the repair, along with differences in surgical methods or technique and the surgeon’s level of proficiency, could all affect the outcomes. These human factors are difficult to standardize and may introduce variability in results that is not easily accounted for, meaning that even under similar experimental conditions, differences in who performed the surgery and how it was done might lead to different recovery outcomes. Fourth, the success of axonal regeneration following surgical repair depends on multiple factors. While graft polarity (the proximal–distal orientation of the graft) is one such factor, other important variables include the specific nerve being repaired and the choice of donor nerve. The timing of surgical intervention relative to the injury is also critical: a prolonged interval between axotomy and reconstruction can lead to chronic denervation of the distal nerve stump and its target muscle, resulting in Schwann cell dysfunction and muscle atrophy, both of which severely impede regeneration. Consequently, the condition of the denervated nerve—encompassing its functional status, the degree of muscle atrophy, and residual conduction capacity—emerges as a key determinant of recovery. Furthermore, technical aspects of the surgical procedure—including the method and angle of nerve transection, graft abutment configuration, and the suturing technique (direct end-to-end coaptation versus interpositional grafting)—can also influence the extent of axonal regeneration achieved. Many of these factors are interrelated, and their interplay likely contributes to the variability in the reported effects of graft orientation across studies. Finally, the sample size and number of studies in this field are relatively limited. We reviewed nine studies comparing the orientation of grafted nerves after nerve transection and performed a meta-analysis on five studies that measured nerve conduction velocity. Although we did not observe a significant overall effect, the confidence intervals were fairly wide (for instance, the 95% CI for the pooled NCV effect spanned approximately −1.52 to +0.37), leaving room for a potential small effect (either positive or negative) that our analysis was not powered to exclude. Further studies are needed to confirm the existence of minor differences.

## 5. Conclusions

This systematic review and meta-analysis provide the first quantitative synthesis of experimental data evaluating the impact of graft orientation (forward vs. reversed) on peripheral nerve regeneration. Unlike earlier narrative reviews, our paper is the first to meta-analyze the experimental data on graft orientation. By pooling available outcome (electrophysiological measure), we provide summary effect estimates with confidence intervals. This statistical synthesis gives a clearer picture of the overall evidence than individual studies can. It demonstrates explicitly how small and inconsistent the overall effect is, information that was not previously available. We adhered to PRISMA guidelines, conducted a risk-of-bias assessment, and identified substantial heterogeneity among included studies—reflecting differences in species, nerve types, and outcome measures. In doing so, we not only clarified existing trends but also highlighted critical evidence gaps, such as the lack of animal studies and virtually no clinical data addressing this question.

In summary, our review organizes the scattered preclinical data on nerve graft polarity into a coherent whole. We find no compelling evidence that graft orientation alone decisively influences regeneration outcomes; any effect is minimal at best. Because the available evidence is limited and heterogeneous, these conclusions are necessarily tentative. Importantly, this work quantifies and clarifies what was previously qualitative or anecdotal, and it highlights where evidence is uncertain. These observations are tentative, derived exclusively from heterogeneous animal studies, and must be interpreted with caution. Nevertheless, by quantifying the current evidence and identifying specific scenarios where graft polarity may matter, this review provides a foundation for future experimental and clinical investigations.

## Figures and Tables

**Table 1:** Characteristics of included studies.

Authors (Publish Year)	Model	Sample Size	Donor Nerve	Recipient Nerve	Observation Period	Assessment Modality	Result
Ansselin AD [14]	Animal (Rats)	40	sciatic nerve	sciatic nerve	2 months	Axon diameter Myelin thickness of axons Cross-sectional area	Reverse
Ansselin AD [15]	Animal (Wistar rats)	62	sciatic nerve	sciatic nerve	12 months	Conduction Velocity Action potential (amplitude) Cross-sectional area Neuron counts Axon diameter Myelin thickness of axons	Reverse
Fujiwara T [18]	Animal (Sprague-Dawley rats)	12	sciatic nerve	sciatic nerve	Not reported	Conduction Velocity Action potential (amplitude) Contraction force of muscle Weight of muscle Neuron count Axon width	Reverse
Lee JM [6]	Animal (Sprague-Dawley rats)	18	facial nerve	facial nerve	4 and 8 weeks	Myelin thickness of axons Conduction Velocity Neuron count	No difference
Nakatsuka H [13]	Animal (Rabbits)	12	peroneal nerve	peroneal nerve	6 months	Conduction Velocity Neuron count Axon density Weight of muscle	No difference
Sotereanos DG [19]	Animal (Rats)	60	sciatic nerve	sciatic nerve	4 months	Sciatic functional index	No difference
Stromberg BV [12]	Animal (Sprague-Dawley rats)	40	sciatic nerve	sciatic nerve	4–6 months	Conduction Velocity Action potential (amplitude)	No difference
Kim JH [11]	Animal (Rabbits)	26	tibial nerve	peroneal nerve	8 weeks	Conduction Velocity Neuron count	No difference
Musa Ergin [20]	Animal (Wistar rats)	30	sciatic nerve	sciatic nerve	12 weeks	Axon count Myelin thickness	No difference

**Table 2:** Comparison of Forward vs. Reverse Nerve Grafting in Animal Nerve Injury Studies.

Feature	Forward Grafting (Orthodromic) 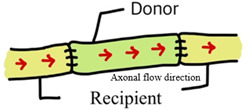	Reverse Grafting (Antidromic) 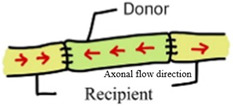
Autograft Orientation	Proximal of graft to proximal of recipient	Distal of graft to proximal of recipient
Direction of Native Axonal Flow	Matches recipient axon direction	Opposes recipient axon direction
Position of Branch Points	Distally oriented in graft	Proximally oriented in graft
Axonal Sprouting Tendency	Axons may divert into distal side branches	Less likely to enter (proximal) branch stumps
Potential Functional Impact	Higher chance of misrouting at branch points	May improve targeting in branched nerves
Animal Models	Wistar rat, Sprague-Dawley rat, Rabbit	Wistar rat, Sprague-Dawley rat
Nerve Types	Facial, sciatic, peroneal	Sciatic

## Data Availability

The original contributions presented in this study are included in the article or Supplementary Materials. Further inquiries can be directed to the corresponding author.

## Supplementary Materials

The following supporting information can be downloaded at: https://www.mdpi.com/article/10.3390/jcm14248885/s1, Table S1: PRISMA checklist, Figure S1: funnel plot of nerve conduction velocity (NCV) [23]. This systematic review was retrospectively registered on the OSF (Open Science Framework). The following are available online at OSF; accessed on 18 November 2025; https://osf.io/a6h8p/overview.
