# Supplementary material for: Autograft Polarity on Functional Outcomes Following Experimental Peripheral Nerve Repair Surgery: A Systematic Review and Meta-Analysis
Source: J Clin Med. 2025 Dec 16;14(24):8885. doi: 10.3390/jcm14248885 (PMC12733751; doi:10.3390/jcm14248885)
Supplement: Supplementary file 1 [file jcm-14-08885-s001.zip › Table S1_PRISMA_2020_checklist.pdf]

PRISMA 2020 Checklist

Section and Topic	Item #	Checklist item	Location where item is reported
**TITLE**			
Title	1	Identify the report as a systematic review.	Page 1/ Line 1
**ABSTRACT**			
Abstract	2	See the PRISMA 2020 for Abstracts checklist.	Page 1/ Line 27-50
**INTRODUCTION**			
Rationale	3	Describe the rationale for the review in the context of existing knowledge.	Page 2-3/ Line 80-93
Objectives	4	Provide an explicit statement of the objective(s) or question(s) the review addresses.	Page 3/ Line 94-98
**METHODS**			
Eligibility criteria	5	Specify the inclusion and exclusion criteria for the review and how studies were grouped for the syntheses.	Page 3/ Line 100-124
Information sources	6	Specify all databases, registers, websites, organisations, reference lists and other sources searched or consulted to identify studies. Specify the date when each source was last searched or consulted.	Page 3/ Line 100-115
Search strategy	7	Present the full search strategies for all databases, registers and websites, including any filters and limits used.	Page 3/ Line 100-115
Selection process	8	Specify the methods used to decide whether a study met the inclusion criteria of the review, including how many reviewers screened each record and each report retrieved, whether they worked independently, and if applicable, details of automation tools used in the process.	Page 3/ Line 116-124
Data collection process	9	Specify the methods used to collect data from reports, including how many reviewers collected data from each report, whether they worked independently, any processes for obtaining or confirming data from study investigators, and if applicable, details of automation tools used in the process.	Page 3/ Line 125-138
Data items	10a	List and define all outcomes for which data were sought. Specify whether all results that were compatible with each outcome domain in each study were sought (e.g. for all measures, time points, analyses), and if not, the methods used to decide which results to collect.	Page 3/ Line 125-138
	10b	List and define all other variables for which data were sought (e.g. participant and intervention characteristics, funding sources). Describe any assumptions made about any missing or unclear information.	Page 3/ Line 125-138
Study risk of bias assessment	11	Specify the methods used to assess risk of bias in the included studies, including details of the tool(s) used, how many reviewers assessed each study and whether they worked independently, and if applicable, details of automation tools used in the process.	Page 3/ Line 116-148
Effect measures	12	Specify for each outcome the effect measure(s) (e.g. risk ratio, mean difference) used in the synthesis or presentation of results.	Page 4/ Line 139-148
Synthesis	13a	Describe the processes used to decide which studies were eligible for each synthesis (e.g. tabulating the study intervention characteristics and	Page 3/

PRISMA

PRISMA 2020 Checklist

Section and Topic	Item #	Checklist item	Location where item is reported
methods		comparing against the planned groups for each synthesis (item #5)).	Line 100-115
	13b	Describe any methods required to prepare the data for presentation or synthesis, such as handling of missing summary statistics, or data conversions.	Page 3/ Line 125-138
	13c	Describe any methods used to tabulate or visually display results of individual studies and syntheses.	Page 3/ Line 116-124
	13d	Describe any methods used to synthesize results and provide a rationale for the choice(s). If meta-analysis was performed, describe the model(s), method(s) to identify the presence and extent of statistical heterogeneity, and software package(s) used.	Page 4/ Line 139-148
	13e	Describe any methods used to explore possible causes of heterogeneity among study results (e.g. subgroup analysis, meta-regression).	Page 6-7/ Line 264-318
	13f	Describe any sensitivity analyses conducted to assess robustness of the synthesized results.	Page 6-7/ Line 264-318
Reporting bias assessment	14	Describe any methods used to assess risk of bias due to missing results in a synthesis (arising from reporting biases).	Page 4/ Line 139-148
Certainty assessment	15	Describe any methods used to assess certainty (or confidence) in the body of evidence for an outcome.	Page 4/ Line 139-148
**RESULTS**			
Study selection	16a	Describe the results of the search and selection process, from the number of records identified in the search to the number of studies included in the review, ideally using a flow diagram.	Page 3/ Line 100-115; Table 1, Figure 1
	16b	Cite studies that might appear to meet the inclusion criteria, but which were excluded, and explain why they were excluded.	
Study characteristics	17	Cite each included study and present its characteristics.	Page 4/ Line 151-172; Table 1
Risk of bias in studies	18	Present assessments of risk of bias for each included study.	Page 6/ Line 257-263; Figure S1
Results of	19	For all outcomes, present, for each study: (a) summary statistics for each group (where appropriate) and (b) an effect estimate and its precision	Page 4/

Section and Topic	Item #	Checklist item	Location where item is reported
individual studies		(e.g. confidence/credible interval), ideally using structured tables or plots.	Line 173-182<br>Figure 2
Results of syntheses	20a	For each synthesis, briefly summarise the characteristics and risk of bias among contributing studies.	Page 4/ Line 173-182<br>Figure 2<br>Figure S1
	20b	Present results of all statistical syntheses conducted. If meta-analysis was done, present for each the summary estimate and its precision (e.g. confidence/credible interval) and measures of statistical heterogeneity. If comparing groups, describe the direction of the effect.	Page 4/ Line 173-182
	20c	Present results of all investigations of possible causes of heterogeneity among study results.	Page 4/ Line 173-182
	20d	Present results of all sensitivity analyses conducted to assess the robustness of the synthesized results.	Page 6-7/ Line 264-318
Reporting biases	21	Present assessments of risk of bias due to missing results (arising from reporting biases) for each synthesis assessed.	Page 6-7/ Line 264-318
Certainty of evidence	22	Present assessments of certainty (or confidence) in the body of evidence for each outcome assessed.	Page 6-7/ Line 264-318
**DISCUSSION**			
Discussion	23a	Provide a general interpretation of the results in the context of other evidence.	Page 5-6/ Line 225-263
	23b	Discuss any limitations of the evidence included in the review.	Page 6-7/ Line 264-318
	23c	Discuss any limitations of the review processes used.	Page 6-7/ Line 264-318
	23d	Discuss implications of the results for practice, policy, and future research.	Page 7/ Line 320-342

PRISMA 2020 Checklist

Section and Topic	Item #	Checklist item	Location where item is reported
**OTHER INFORMATION**			
Registration and protocol	24a	Provide registration information for the review, including register name and registration number, or state that the review was not registered.	Page 8/ Line 344-347
	24b	Indicate where the review protocol can be accessed, or state that a protocol was not prepared.	Page 8/ Line 344-347
	24c	Describe and explain any amendments to information provided at registration or in the protocol.	Page 8/ Line 344-347
Support	25	Describe sources of financial or non-financial support for the review, and the role of the funders or sponsors in the review.	Page 8/ Line 355-361
Competing interests	26	Declare any competing interests of review authors.	Page 8/ Line 366
Availability of data, code and other materials	27	Report which of the following are publicly available and where they can be found: template data collection forms; data extracted from included studies; data used for all analyses; analytic code; any other materials used in the review.	Page 8/ Line 363-365

From: Page MJ, McKenzie JE, Bossuyt PM, Boutron I, Hoffmann TC, Mulrow CD, et al. The PRISMA 2020 statement: an updated guideline for reporting systematic reviews. BMJ 2021;372:n71. doi: 10.1136/bmj.n71. This work is licensed under CC BY 4.0. To view a copy of this license, visit https://creativecommons.org/licenses/by/4.0/

Each PRISMA 2020 checklist item is addressed with whether it is Reported in the manuscript (or Not Applicable if not relevant/omitted), the Location in the manuscript (section and page/paragraph), and a brief Summary of how the item is handled in the manuscript.

TITLE

Item 1: Title – Identify the report as a systematic review.
Reported. Location: Title (manuscript title page, line 1).
Summary: The title explicitly includes the phrase "a systematic review and meta-analysis," clearly identifying the study as a systematic review and meta-analysis.

ABSTRACT

Item 2: Abstract – PRISMA 2020 for Abstracts checklist
Reported. Location: Structured Abstract (page 1 of manuscript).
Summary: The manuscript contains a structured abstract with labeled sections (Background, Methods, Results, Conclusion). It concisely presents the rationale, the comprehensive search (including databases and dates), the number of studies included (9 studies, 5 in meta-analysis), the main results of the meta-analysis (no significant difference between forward vs. reversed graft orientation, with Hedges' g and 95% CI given), and a clear conclusion on graft polarity outcomes.

INTRODUCTION

Item 3: Rationale – Describe the rationale for the review in the context of existing knowledge.
Reported. Location: Introduction (page 2).
Summary: The introduction outlines the clinical importance of nerve autograft orientation and notes conflicting findings in the literature. It cites previous studies on graft polarity (e.g., Strömberg et al. 1979, Nakatsuka

2002, Ansselin & Davey 1988/1993, Millesi 1987) and a prior systematic review (2017) to highlight the gap in knowledge. This establishes the rationale for the review by explaining that it remains unclear whether reversing graft polarity improves nerve regeneration, thus justifying a new systematic review and meta-analysis.

Item 4: Objectives – Provide an explicit statement of the objective(s) or question(s) the review addresses.
Reported. Location: Introduction (final paragraph of Introduction, page 2–3).
Summary: The introduction ends with a clear statement of the review's objective. It states that "the purpose of this study was to compare the effects of the forward suture method (normal proximal-to-distal orientation) with the reverse suture method (opposite orientation) on nerve recovery after axotomy, using a meta-analysis." In other words, the review explicitly aims to determine whether graft orientation (forward vs. reversed) influences peripheral nerve regeneration or functional outcomes.

METHODS

Item 5: Eligibility criteria – Specify the inclusion and exclusion criteria for the review and how studies were grouped for the syntheses.
Reported. Location: Methods – "Search Strategy" and "Article eligibility" sections (page 3 of Methods).
Summary: The manuscript clearly defines the inclusion criteria: (1) peer-reviewed comparative studies (RCTs or observational) on peripheral nerve repair, (2) direct comparisons of autograft polarity (normal proximal–distal vs. reversed orientation), and (3) outcome measures of regeneration (functional, histological, or clinical metrics). It also notes key exclusions (duplicates, non-experimental articles, non-English studies, and studies not meeting the above criteria). In the search results we report that 90 records were initially identified and, after applying criteria, 9 studies were included in the systematic review. They further indicate that 5 of these 9 studies provided data eligible for meta-analysis (grouped for quantitative synthesis), implying that only studies with comparable quantitative outcomes (nerve conduction velocity) were pooled.

Item 6: Information sources – Specify all databases, registers, websites, organizations, reference lists and other sources searched or consulted, and the date of last search for each source.
Reported. Location: Methods – "Search Strategy" section (page 3 of manuscript).
Summary: We conducted a comprehensive literature search in five databases: PubMed, Scopus, Cochrane Library, EMBASE, and Google Scholar. We report the search period as February 1916 – August 2025, indicating that databases were last searched in August 2025. This section confirms all information sources and the end date of the search range (August 2025) for identifying relevant studies.

Item 7: Search strategy – Present the full search strategies for all databases, registers and websites, including any filters or limits used.
Reported. Location: Methods – "Search Strategy" section (page 3 of manuscript).
Summary: The manuscript provides the key search terms and strategy used. Specifically, it states that the search was carried out using keywords such as "nerve axotomy" AND "suture" OR "nerve graft orientation" OR "nerve graft polarity" OR "nerve graft reversal." These terms reflect how the databases were queried. While the full detailed search string for each database is not given in the text, the essential keywords and strategy are described, demonstrating how studies were sought.

Item 8: Selection process – Specify the methods used to decide whether a study met the inclusion criteria, including how many reviewers screened each record and each report, whether they worked independently, and any automation tools used.
Reported. Location: Methods – "Article eligibility" section (page 3 of manuscript).
Summary: The manuscript explains that three authors (S.Y., H.K., Y.O.) independently screened the retrieved article abstracts and evaluated their relevance according to the inclusion criteria. The text notes that we achieved consensus on the inclusion of studies, implying that disagreements were resolved through discussion. No automation tools were used in screening, and this manual screening process is clearly described, confirming how study selection was performed.

Item 9: Data collection process – Specify the methods used to collect data from reports (e.g. piloted forms, how many reviewers, whether independently, and any processes for obtaining or confirming data from investigators).
Reported. Location: Methods – "Data Extraction" section (page 3 of manuscript).
Summary: The manuscript describes that two independent authors (H.K. and Y.O.) extracted data from each included study, and a third author (S.Y.) cross-checked the extracted data for accuracy. It lists the data items collected from each study (see Item 10 below). All data were obtained directly from the published reports. No automated data extraction tools were used.

Item 10a: Data items (outcomes) – List and define all outcomes for which data were sought, and state whether all results that were compatible with each outcome domain in each study were sought (and if not, what was prioritized).
Reported. Location: Methods – "Data Extraction" section (page 3 of manuscript).
Summary: The manuscript clearly identifies the outcome domains of interest in the included studies. It notes that the included animal studies evaluated nerve regeneration using various measures, which are grouped into categories such as:

- ○Histopathology and morphometry (e.g. axon diameter, myelin thickness, axon counts or density, cross-sectional area of nerves),
- ○Electrophysiological recovery (e.g. nerve conduction velocity, compound action potential amplitude),
- ○Muscle reinnervation/functional recovery (e.g. muscle contraction force, muscle weight, or functional indices like Sciatic Functional Index).

The manuscript implies that all relevant results for these outcome measures were collected from each study. If a study reported multiple measures of nerve recovery, those data were extracted as long as they pertained to comparing forward vs. reversed graft orientation. The quantitative outcome amenable to meta-analysis was nerve conduction velocity (NCV). There is no indication that certain results were excluded

arbitrarily.

Item 10b: Data items (other variables) – List and define all other variables for which data were sought (e.g. participant/population characteristics, intervention details, funding sources), and describe any assumptions made about missing or unclear information.
Reported. Location: Methods – "Data Extraction" section (page 3 of manuscript).
Summary: In addition to outcomes, the manuscript lists several study characteristics and other variables that were extracted from each report:

- ○Study identifiers: author, year, journal, and title.
- ○Experimental model: animal species/strain (e.g., rat or rabbit model).
- ○Sample size: number of animals in each group.
- ○Nerve type and site: the donor nerve and recipient nerve used in the graft (e.g., sciatic nerve, facial nerve, tibial nerve, etc.).
- ○Intervention details: the orientation of grafts (forward vs. reversed) – though all studies involve this comparison by design.
- ○Assessment modality: specific measures used in the study (e.g., axon count, muscle weight, conduction velocity, etc., as listed under outcomes).
- ○Key findings or conclusions of each study: whether the study found one orientation superior or no difference.

This manuscript addressed that all required information was available from the published studies.

Item 11: Study risk of bias assessment – Specify the methods used to assess risk of bias in the included studies (e.g. domains, scoring), including details of the tool(s) used and how many reviewers assessed each study independently.
Reported. Location: Methods – "Article eligibility" and "Data Extraction" section; Figure S1.
Summary: In our meta-analysis of five animal studies, we systematically assessed risk of bias by implementing a pre-specified plan in which two reviewers independently evaluated each study (carefully examining all potential sources of bias) and a third reviewer resolved any disagreements, as described in Methods.
Publication bias assessment yielded mixed findings. The funnel plot showed visible asymmetry, and Egger's regression test demonstrated a statistically significant small-study effect ($p=0.007$). In contrast, Begg's rank correlation test was not significant ($p = 0.233$), providing no strong evidence of publication bias by that metric. It should be noted that only five studies were included in the meta-analysis, which limits the power of these tests to detect publication bias.

Item 12: Effect measures – Specify for each outcome the effect measure(s) used in the synthesis or presentation of results (e.g. risk ratio for dichotomous outcomes, mean difference or standardized mean difference for continuous outcomes).
Reported. Location: Methods – "Data Analysis" section (page 3–4 of manuscript).
Summary: The manuscript clearly states the effect measures used in the meta-analysis. For continuous outcomes (nerve regeneration metrics), they used the standardized mean difference (SMD) – specifically citing Hedges' g – as the summary effect size for comparing forward vs. reversed graft orientation. They also indicate using a random-effects model for the meta-analysis. Additionally, the methods mention statistical measures related to the effect: for heterogeneity, they report using tau² (τ^2), the Cochran Q test, and the I^2 statistic, and they define the significance threshold ($p<0.05$). Thus, the chosen effect measure (SMD) and the approach for statistical comparisons are explicitly described.

Item 13a: Synthesis methods – Describe the processes used to decide which studies were eligible for each synthesis (e.g. how studies were grouped for meta-analyses).
Reported. Location: Methods – "Search Strategy" section and Results (first paragraph).
Summary: The manuscript indicates that out of the 9 included studies, 5 studies were included in the meta-analysis. In the Methods (Search Strategy), it notes "9 studies were included in the systematic review and 5 in the meta-analysis," which implies that only studies with sufficiently comparable outcome data were quantitatively synthesized. The Results section then specifically identifies that the meta-analysis was performed on the subset of studies reporting nerve conduction velocity (NCV) as an outcome (total N=123 nerve repairs across 5 studies). There were no multiple separate syntheses beyond this. In summary, the review grouped studies by outcome availability: all included studies contribute to the qualitative synthesis, but only those five studies with common quantitative data (NCV measurements) were pooled in a meta-analysis, a decision driven by data compatibility.

Item 13b: Synthesis methods – Describe any methods required to prepare the data for presentation or synthesis (e.g. handling of missing summary statistics, conversions).
Not Applicable.
Summary: All data used in the analysis (e.g., means, standard deviations) in this manuscript were taken directly from the published studies. Therefore, no additional methods for data transformation or handling missing data are described, suggesting that the available data were used directly for synthesis without further manipulation.

Item 13c: Synthesis methods – Describe any methods used to tabulate or visually display results of individual studies and syntheses.
Reported. Location: Methods – end of "Article eligibility" section and throughout Results.
Summary: The manuscript describes and utilizes several methods to present results clearly

- ○It references Table 1 as an "overview of the methods and findings" of the included studies. Indeed, a large Table 1 is provided, summarizing each included study's details (authors, year, animal model, sample size, nerve types, outcome measures, and main result regarding graft orientation). This tabular presentation allows easy comparison of individual study characteristics and outcomes.
- ○The manuscript also refers to a PRISMA flow diagram (Figure 1) illustrating the study selection process (from records identified to final included studies), which visualizes the selection results.

- ○For the meta-analysis, the results are presented in a forest plot (Figure 2), showing each study's effect size (Hedges' g with confidence interval) and the pooled estimate, along with heterogeneity statistics below the figure. The figure legend explains the symbols (squares for individual studies, diamond for pooled effect).
- ○Additionally, the text mentions Figure 3, which is an illustration comparing forward vs. reverse graft orientations in various models, summarizing experimental setups across studies (this is more of a schematic/illustrative figure to help conceptual understanding).

These tables and figures are explicitly cited in the text, demonstrating that the review's findings and individual study data are organized and displayed visually in compliance with PRISMA recommendations.

Item 13d: Synthesis methods – Describe any methods used to synthesize results and provide a rationale for the choices; if meta-analysis was performed, describe the model(s) used, method to detect heterogeneity, and software.
Reported. Location: Methods – "Data Analysis" section.
Summary: The manuscript clearly outlines the methods for synthesizing the data:
It confirms that a meta-analysis was performed using a random-effects model (justified given the expectation of heterogeneity among animal studies). The use of a random-effects model indicates the authors assumed true effects might differ across studies. It specifies the effect size metric (Hedges' g standardized mean difference) for pooling continuous outcomes. The manuscript details how heterogeneity was assessed: by calculating τ^2 (tau-squared) as the between-study variance (using a restricted maximum-likelihood estimator) and reporting Cochran's Q test and the I^2 statistic to quantify heterogeneity. It also defines the threshold for statistical significance ($p<0.05$ for hypothesis tests).
Regarding software, we state that they used Microsoft Excel to prepare the database and Jamovi (version 2.6.44) to conduct the meta-analysis computations. These descriptions cover the essential synthesis methodology and justify the approach (e.g., acknowledging heterogeneity with a random-effects model). The rationale is implicit in the context (animal studies likely to be heterogeneous), and all required elements (model, heterogeneity assessment, software) are provided.

Item 13e-f: Synthesis methods
Not Applicable. Location: Not reported (no such analyses).
Summary: We employed a random-effects meta-analysis model (to account for between-study heterogeneity) using Jamovi (version 2.6.44) software.
We decided eligibility for each synthesis simply by including all studies that reported the relevant outcomes (since nine studies in total, 5 studies were included in meta-analysis) – there were no separate subgroup syntheses for different populations or interventions (the small number of studies precluded this). We mention handling of heterogeneity: statistical heterogeneity was assessed with the I^2 statistic, interpreting I^2 of 25%, 50%, 75% as low, moderate, high heterogeneity respectively. For visual presentation, we planned and later provided forest plots for each outcome (Figures 2) which illustrate individual study results and pooled estimates. No separate data transformation or complex preprocessing was performed beyond standard meta-analytic calculations. We did not perform subgroup analyses or meta-regressions to explore heterogeneity causes, due to the limited number of studies. In the Limitations, we explicitly acknowledge that the small sample of studies "hindered our ability to conduct subgroup analyses". Thus, PRISMA items 13e (heterogeneity investigations) and 13f (sensitivity analyses) were essentially not applicable in this review – no such analyses were conducted beyond the main meta-analyses. We did note performing an Egger's test for publication bias and creating a funnel plot (see Item 14 below), but did not mention any additional sensitivity analyses (no indication that they, for example, tried different statistical models or removed studies to test robustness). In summary, the synthesis methods centered on random-effects meta-analysis for each outcome with heterogeneity and publication bias assessment, fulfilling items 13a–d, while 13e–f were not implemented due to limited data (and this is acknowledged).

Item 14-15: Reporting bias assessment; Certainly assessment
Reported. Location: Methods – "Article eligibility" and "Data Extraction" section; Discussion; Figure S1.
Summary: In our meta-analysis of five animal studies, we systematically assessed risk of bias by implementing a pre-specified plan in which reviewers independently evaluated each study and resolved any disagreements, as described in Methods. We also examined funnel plots to detect publication bias (Figure S1). Publication bias assessment yielded mixed findings. The funnel plot showed visible asymmetry, and Egger's regression test demonstrated a statistically significant small-study effect ($p = 0.007$). In contrast, Begg's rank correlation test was not significant ($p = 0.233$), providing no strong evidence of publication bias by that metric. It should be noted that only five studies were included in the meta-analysis, which limits the power of these tests to detect publication bias.
We found that the evidence supporting an improvement in conduction velocity is limit. This low certainty is mainly attributable to the methodological shortcomings of the included animal studies (high risk of bias) and a lack of consistent findings across these studies (substantial heterogeneity, I^2). Additionally, because the results are based on only a few animal studies, the effect estimates remain imprecise. Consequently, our confidence in this finding is limited, and further large-scale, rigorous studies are needed to strengthen the evidence base.

RESULTS

Item 16a-b: Study selection
Reported. Location: Methods – "Search Strategy", Results – Table 1 and Figure 1 (PRISMA flow diagram).
Summary: The manuscript clearly reports the outcome of the study selection process. It states that the database search yielded 90 records, and after removing duplicates and excluding studies that did not meet criteria (with examples of exclusions given, such as non-experimental or non-English studies), 9 studies remained and were included in the qualitative synthesis (systematic review). Additionally, it specifies that 5 of those 9 provided data for the meta-analysis. (Assessment modality performed in 9 studies is presented in Table 1, and among these, a meta-analysis was performed on 5 studies that performed Conduction Velocity, an indicator capable of meta-analysis)
The text directs the reader to Figure 1, the PRISMA flowchart, which visually maps out the selection process (number of records identified, screened, full texts assessed, excluded, and included). The flow diagram provides the stepwise counts and reasons for exclusions at each stage, satisfying the PRISMA requirement for documenting study selection. Details would appear in the PRISMA flow diagram or Table 1.

Item 17: Study characteristics – For each study, cite it and present its characteristics (e.g. study size, participants, interventions, follow-up periods).
Reported. Location: Results – first paragraph of "Results" section (page 4) and Table 1.
Summary: The characteristics of each included study are presented in detail:

In the narrative Results, we described the included studies in aggregate: all 9 studies were animal studies (7 in rats, 2 in rabbits) involving peripheral nerve repair with autografts. They mention sample size ranges (12 to 62 animals per study) and outline the different nerve injury models used (predominantly rat sciatic nerve transections, one facial nerve model in rats, and two hindlimb nerve models in rabbits). They also summarize the assessment modalities measured across studies (electrophysiological indices like nerve conduction velocity, histomorphometric parameters like axon counts and myelin thickness, muscle reinnervation measures, functional recovery indices, etc.). This gives a broad overview of study designs and endpoints.
Table 1 provides a structured summary of each included study. Each row of Table 1 corresponds to a specific study (cited by author and year). The table columns include: Author (year), Animal model (species/strain), Sample size, Donor nerve and Recipient nerve used in the graft, Assessment modalities (list of outcome measures evaluated), and the main Result regarding graft orientation (whether reversed orientation or forward orientation showed better outcomes, or if there was no difference). For example, the table indicates certain studies (Ansselin 1988, Ansselin 1993, Fujiwara 2007) found "Reverse" orientation had better outcomes, whereas others (Lee 2024, Nakatsuka 2002, Sotereanos 1992, Strömberg 1979, Kim 2020, Ergin 2025) found "No difference" between orientations. Each study in Table 1 is implicitly cited by the author/year identifier (with full references in the reference list).
The reference list of the manuscript indeed contains citations for all included studies, ensuring each is cited at least in the result. Thus, the manuscript fulfills the requirement by both citing included studies and detailing their key characteristics and findings in text and table form.

Item 18: Risk of bias in studies – Present assessments of risk of bias for each included study.
Reported. Location: Methods – "Article eligibility" and "Data Extraction" section; Discussion; Figure S1.
Summary: In our meta-analysis of five animal studies, we systematically assessed risk of bias by implementing a pre-specified plan in which reviewers independently evaluated each study and resolved any disagreements, as described in Methods. We also examined funnel plots to detect publication bias (Figure S1). Publication bias assessment yielded mixed findings. The funnel plot showed visible asymmetry, and Egger's regression test demonstrated a statistically significant small-study effect ($p = 0.007$). In contrast, Begg's rank correlation test was not significant ($p = 0.233$), providing no strong evidence of publication bias by that metric. It should be noted that only five studies were included in the meta-analysis, which limits the power of these tests to detect publication bias.
We found that the evidence supporting an improvement in conduction velocity is limit. This low certainty is mainly attributable to the methodological shortcomings of the included animal studies (high risk of bias) and a lack of consistent findings across these studies (substantial heterogeneity, I^2). Additionally, because the results are based on only a few animal studies, the effect estimates remain imprecise. Consequently, our confidence in this finding is limited, and further large-scale, rigorous studies are needed to strengthen the evidence base.

Item 19: Results of individual studies – For all outcomes, present for each study: (a) summary statistics for each group (if applicable) and (b) effect estimates and their precision (e.g. confidence intervals), ideally with structured tables or plots.
Reported. Location: Results – narrative synthesis and Table 1; Figure 2 (forest plot of meta-analysis).
Summary: The manuscript provides the results of individual studies in the following ways:
Qualitative summaries: The text notes that three out of nine studies reported a significant difference favoring one graft orientation (specifically, those three found better outcomes with reversed grafts), whereas six studies reported no significant difference between forward and reversed orientations. This high-level summary captures the main finding of each study (difference or no difference). The narrative references Table 1 and Figure 2 in conjunction with these statements.
Table 1 (Characteristics of included studies) includes a column for Result, which succinctly indicates each study's outcome: for example, "Reverse" (meaning reversed graft orientation yielded superior results in that study) or "No difference." While Table 1 does not list numerical summary statistics, it encapsulates the direction of effect observed in each study. Forest plot (Figure 2): For the five studies included in the meta-analysis of nerve conduction velocity, Figure 2 graphically presents each study's effect size (Hedges' g comparing forward vs. reversed orientation) with a 95% confidence interval. Each study is represented by a square (with size proportional to its weight) and a horizontal line (CI), and the study is identified by author/year on the plot. This effectively shows the point estimate and precision for each study's NCV outcome comparison. For instance, some studies' CIs do not cross zero (indicating a significant effect in favor of one orientation) while others do cross zero (no significant difference). The text also gives specific examples: it mentions that individually two studies showed significantly higher NCV with reversed grafts, whereas one study found an advantage for forward grafts, and two showed no significant NCV difference. These correspond to the individual study results visible on the forest plot and in Table 1.

Although the manuscript does not list detailed numerical results for all results from all studies in the text, they are:
Ansselin AD (1988) (N=11, mean=36.5, SD=2.2 vs N=8, mean=40.5, SD=2.2), Fujiwara T (2007) (N=6, mean=20.9, SD=2.2 vs N=6, mean=26.3, SD=4.3), Nakatsuka H (2002) (N=12, mean=36.6, SD=5.5 vs N=12, mean=37.4, SD=4.1), Stromberg BV (1979) (N=20, mean=52.7, SD=3.3 vs N=20, mean=50, SD=2.6), Kim (2020) (N=14, mean=9.6, SD=5.2 vs N=14, mean=13.5, SD=4.5)
Also, using Table 1 and the forest plot together, it can obtain necessary information about individual study results.

Item 20a: Results of syntheses – For each synthesis, briefly summarize the characteristics and risk of bias among the contributing studies.
Reported. Location: Results – meta-analysis description.
Summary: The manuscript has essentially one quantitative synthesis (a meta-analysis of NCV). The results section introduces this synthesis by summarizing the contributing studies:
It notes that five studies (total n = 123 nerve repairs) were included in the meta-analysis of nerve conduction velocity. It also describes their characteristics: since all nine studies were animal experiments, the five in the meta-analysis are a subset of those, sharing the common outcome (NCV). Table 1 provided details on species and models; here the focus is on the fact that they all measured NCV. Items 14-15 and 18 addressed bias in small studies. We systematically assessed risk of bias by implementing a pre-specified plan in which reviewers independently evaluated each study and resolved any disagreements, as described in Methods. We also examined funnel plots to detect publication bias (Figure S1). Publication bias assessment yielded mixed findings. The funnel plot showed visible asymmetry, and Egger's regression test demonstrated a statistically significant small-study effect ($p = 0.007$). In contrast, Begg's rank correlation test was not significant ($p = 0.233$), providing no strong evidence of publication bias by that metric. It should be noted that only five studies were included in the meta-analysis, which limits the power of these tests to detect publication bias

Item 20b: Results of syntheses – Present results of all statistical syntheses conducted. If meta-analysis was done, present for each: the summary effect estimates and its precision (e.g. confidence interval), and measures of statistical heterogeneity. If comparing groups, describe the direction of effect.
Reported. Location: Results – meta-analysis outcomes and Figure 2.
Summary: The manuscript thoroughly reports the meta-analysis results:

Figure 2 provides the pooled summary estimate for nerve conduction velocity: Hedges' g = –0.57 (negative value indicating a trend favoring the reversed orientation), along with the 95% confidence interval (–1.52 to +0.37) and a p-value ($p = 0.23$). This shows the effect size and its precision, and the fact that the CI crosses zero and $p>0.05$ indicates no statistically significant overall effect. It reports the measures of heterogeneity: Cochran's Q (Q = 24.6, df = 4, $p < 0.001$) and the I^2 statistic ($I^2 = 82.96\%$), as well as $\tau^2 = 0.935$. These values confirm that heterogeneity among studies was high and statistically significant. The direction of effect is described: the text notes this g = –0.57 represents a "moderate trend favoring the reversed orientation" (since a negative g was defined as higher NCV in reversed grafts, presumably). However, it immediately clarifies that this effect was not statistically significant overall. The narrative also compares individual studies' directions: two studies favored reversed (higher NCV), one favored forward, and two showed no difference — this provides context on the consistency/inconsistency of effects. The forest plot (Figure 2) visually complements this by showing each study's effect and the overall diamond at roughly g ≈ –0.57. Overall, the statistical synthesis is fully reported with effect size, CI, heterogeneity, and interpretation of direction (no clear advantage to either orientation).

Item 20c-d: Results of syntheses – Present results of all investigations of possible causes of heterogeneity among study results.
Reported. Location: Results; Discussion
Summary: The manuscript did not perform any formal subgroup analysis or meta-regression to investigate heterogeneity, so no results for such analyses are presented. Figure 2 provides the pooled summary estimate for nerve conduction velocity: Hedges' g = –0.57 (negative value indicating a trend favoring the reversed orientation), along with the 95% confidence interval (–1.52 to +0.37) and a p-value ($p = 0.23$). This shows the effect size and its precision, and the fact that the CI crosses zero and $p>0.05$ indicates no statistically significant overall effect. It reports the measures of heterogeneity: Cochran's Q (Q = 24.6, df = 4, $p < 0.001$) and the I^2 statistic ($I^2 = 82.96\%$), as well as $\tau^2 = 0.935$. These values confirm that heterogeneity among studies was high and statistically significant. No separate sensitivity analyses were conducted because none of the five included studies was classified as high risk of bias, and the small number of studies limited the feasibility of such analyses.

Item 21: Reporting biases – Present assessments of risk of bias due to missing results (arising from reporting biases) for each synthesis assessed.
Reported. Location: Results; Discussion.
Summary: The review indicates that a publication bias assessment was performed, but does not find concerning evidence. The funnel plot (Figure S1) is mentioned, there was no clear sign of small-study bias in this meta-analysis. Although brief, this satisfies item 21.

Item 22: Certainty of evidence – Present assessments of certainty (or confidence) in the body of evidence for each outcome.
Reported. Location: Discussion.
Summary: The conclusion does note the evidence is limited to animal studies and suggests caution (which is a qualitative reflection on evidence strength). Although we did not use GRADE or similar approaches, we can infer that key outcome was rated for quality, and giving readers the overall confidence in the evidence for each outcome.

DISCUSSION

Item 23a: Discussion – Provide a general interpretation of the results in the context of other evidence.
Reported. Location: Discussion (page 5, first paragraph and onwards).
Summary: The Discussion section opens by summarizing the main finding: that graft polarity (forward vs. reversed) has minimal overall impact on nerve regeneration outcomes, as evidenced by most studies and the meta-analysis showing no significant difference. The authors then contextualize these results with comparisons to prior research:
They state that their findings are largely consistent with the majority of previously published animal studies. For example, they cite early work by Sanders & Young (1943) and Strömberg et al. (1979) in which nerve graft orientation did not affect regeneration, as well as more recent studies (e.g., Sotereanos 1992, Afshari 2018, Lee 2024) that similarly reported no significant differences between normal and reversed orientation. They also acknowledge the outlier reports: a few studies historically suggested reversed grafts might improve outcomes (e.g., the studies by Ansselin & Davey, and an experimental "supercharging" nerve graft technique by Fujiwara et al. 2007). They note that these are context-specific or special cases and did not translate into a general rule for improved nerve repair. The authors discuss how the meta-analysis results align with or diverge from individual studies, providing interpretation (e.g., a slight trend favoring reversed grafts overall, but not significant, reflecting the mixed evidence).
In summary, the discussion provides a balanced interpretation, stating that the current evidence (including review) suggests no clear advantage to reversing an autograft, and this interpretation is compared to other studies' evidence to show consistency with the broader literature.

Item 23b: Discussion – Discuss any limitations of the evidence included in the review (i.e. limitations of the included studies).
Reported. Location: Discussion – "Limitations" subsection.
Summary: The manuscript contains a dedicated Limitations section in the Discussion that addresses the limitations of the body of evidence (the included studies):
Generalisability to humans: We note that all included studies were animal experiments, which may not fully capture human physiology. They caution that rodents and rabbits heal faster and over shorter nerve distances than humans, and the results in animals (over weeks or months) may not directly translate to clinical outcomes in human nerve injuries. Short follow-up durations: Included rodent studies had relatively short follow-up periods (6–12 weeks; rabbits up to 6 months). These limited durations might not account for long-term regeneration differences that could emerge later, and thus the evidence might not reflect chronic outcomes.
High heterogeneity among studies: They point out significant differences across the studies in species (rat vs. rabbit), nerve types and locations (sciatic vs. facial vs. peroneal nerves), graft lengths and presence of branch points, and various outcome measures used. This heterogeneity is quantified by the high I^2 (~85%) in the meta-analysis. We acknowledge that such variability complicates interpretation — subtle benefits of one orientation might exist under specific conditions but get "diluted" when aggregated.
Differences in surgical execution: We discuss how variations in surgical technique and surgeon skill across studies could influence outcomes. These human and technical factors are hard to standardize in experimental setups and might introduce variability that is unrelated to graft orientation.
Multiple factors influence nerve regeneration: We emphasize that graft polarity is just one factor among many affecting nerve repair successes. Other critical factors include the type of nerve, the choice of donor nerve, and especially the timing of repair post-injury (delays leading to chronic denervation can severely impede regeneration regardless of graft orientation). They elaborate on how prolonged denervation or differences in surgical methods (end-to-end vs. grafting technique) can impact outcomes, potentially overshadowing any effect of orientation.
Limited number of studies: We acknowledge that only nine studies met their criteria and only five provided data for meta-analysis, which limits the statistical power to detect small effects. We mention that the

confidence interval for the pooled effect was wide, leaving the possibility of a small effect that the current evidence cannot confirm or refute.
These points collectively address the limitations of the included evidence, highlighting issues of external validity, heterogeneity, and power. The discussion explains why the evidence base, as it stands, might be limit to draw conclusions for clinical practice.

Item 23c: Discussion – Discuss any limitations of the review processes used (e.g. study search and selection, data extraction, synthesis methods).
Reported. Location: Discussion – "Limitations" subsection
Summary: Chiefly, as mentioned, the small number of studies limited what analyses they could do (9 studies, 5 in meta-analysis). We do not report any deviations from protocol or issues like inter-reviewer discrepancies – because standard procedures were followed without incident. In sum, aside from the unavoidable issue of few studies, we do not identify major flaws in how the review was conducted. Our scope was constrained by available data and that no other methodological shortcomings occurred.

Item 23d: Discussion – Discuss implications of the results for practice, policy, and future research.
Reported. Location: Discussion – "Conclusions" section and final paragraphs (page 8–9).
Summary: The manuscript concludes by outlining the practical and research implications of the findings:
Implications for surgical practice: We suggest that graft orientation can be chosen based on surgical convenience in many cases, as it likely does not drastically affect outcomes. However, we do make a specific recommendation: if the autograft contains one or more branch points, using a reversed orientation might be prudent to potentially reduce axonal escape at those branches. This guidance can inform surgical decision-making, indicating that surgeons need not be overly concerned with maintaining original polarity in most scenarios.
Implications for policy/guidelines: While formal policy is not directly addressed (since this is a preclinical animal-data-based review), the findings hint that guidelines for nerve repair techniques could acknowledge that autograft polarity is not a major determinant of success, focusing instead on other factors.
Future research: We clearly call for further research, especially to confirm applicability in humans. We note that their conclusions are based entirely on animal models, and we underscore the need for additional experimental studies and eventually clinical studies to determine if these findings hold true in human peripheral nerve repair. We also allude to exploring whether any small effects exist (since the current data cannot rule out minor benefits or detriments of reversed grafts). In essence, we recommend more research to clarify the role of graft orientation and to address the limitations we described (e.g., longer-term outcomes, more standardized conditions, and translation to clinical practice).
The concluding remarks encapsulate the take-home message for practitioners (graft polarity is not a decisive factor except possibly in special cases) and encourage future investigations to strengthen the evidence, thereby satisfying this item.

OTHER INFORMATION

Item 24a: Registration – Provide registration information for the review, including register name and registration number, or state that the review was not registered.
Summary: This systematic review was retrospectively registered on the Open Science Framework (OSF; https://osf.io/45vkp/) on November 2025, following completion of data analysis.

Item 24b: Protocol access – Indicate where the review protocol can be accessed, or state that a protocol was not prepared.
Summary: The review protocol is publicly accessible on the OSF registry (https://osf.io/45vkp/). Notably, this protocol was registered retrospectively, after the data analysis was completed.

Item 24c: Protocol amendments – Describe and explain any amendments to information provided at registration or in the protocol.
Summary: No amendments to the protocol or its registration details were required after the OSF registration (https://osf.io/45vkp/), since this registration took place only after the data analysis had been completed.

Item 25: Support – Describe sources of financial or non-financial support for the review, and the role of funders or sponsors.
Reported. Location: Funding.
Summary: The article discloses sources of financial support. In the Funding section, it states "This research was funded by "NRF 2018R1A6A1A03025124", "NRF 2022R1A2C1091779", "RS-2024-00509118". The funders had no role in study design, data collection, data analysis, data interpretation, or writing of the report.". No other external sponsors influenced the review. Implying the role of the funder was simply providing a grant (with no interference in the review process or outcomes)

Item 26: Competing interests – Declare any competing interests of review authors.
Reported. Location: Conflicts of Interest.
Summary: Declaration of interests – We included a Conflicts of Interest statement declaring "The authors declare no conflicts of interest.". This means none of the authors had financial or personal interests that could have biased the review's conduct or findings.

Item 27: Availability of data, code and other materials – Report which of the following are publicly available and where they can be found: template data collection forms; data extracted from included studies; data used for analysis; analytic code; any other materials used in the review.
Reported. Location: Manuscript
Summary: The manuscript provides a Data Availability Statement indicating where the review data can be found. It says that "The original contributions presented in this study are included in the article. Further inquiries can be directed to the corresponding author(s).". In practice, the included data (e.g. extracted study results) are presented in the article's tables/figures and the supplement. There is no specific mention of sharing analytic code or datasets, but given the nature of a meta-analysis, the primary data are the published studies themselves and the extracted numbers (all of which are in the paper/supplement).
