# Supplementary figures and images for: Autograft Polarity on Functional Outcomes Following Experimental Peripheral Nerve Repair Surgery: A Systematic Review and Meta-Analysis
Source: J Clin Med. 2025 Dec 16;14(24):8885. doi: 10.3390/jcm14248885 (PMC12733751; doi:10.3390/jcm14248885)

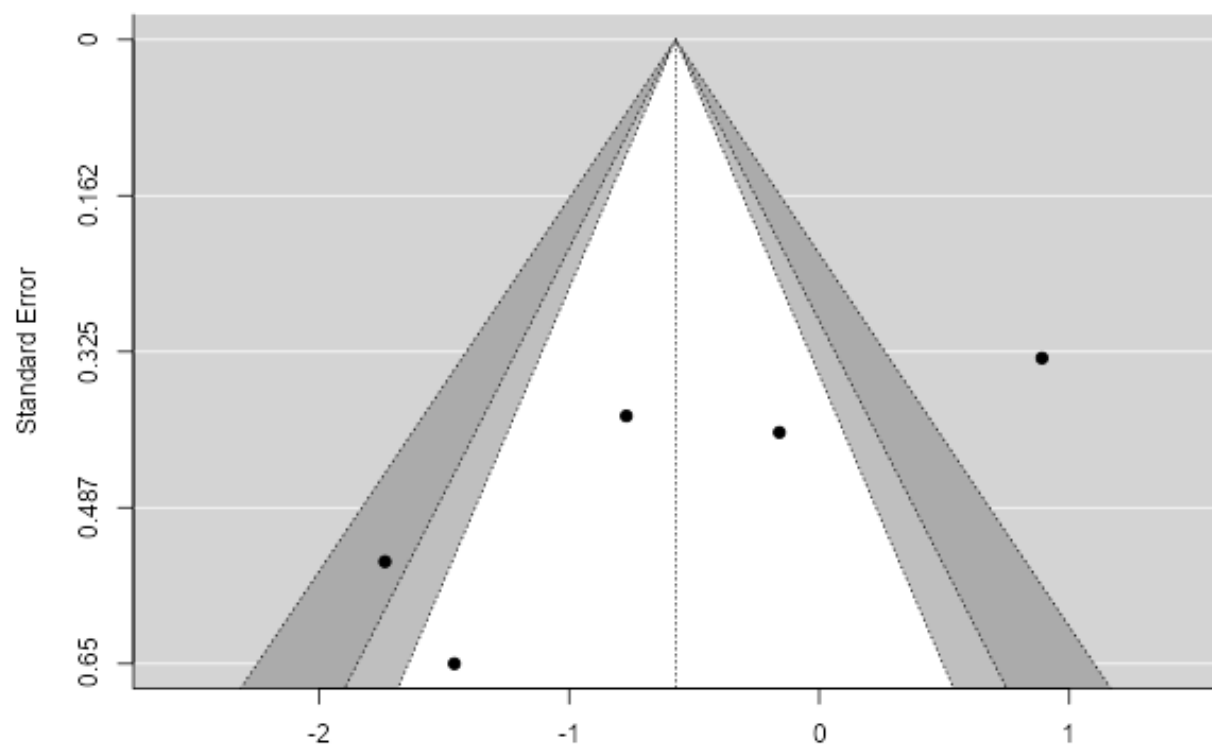

Figure S1. Funnel plots of nerve conduction velocity (NCV).

Supplement: Supplementary file 1 [file jcm-14-08885-s001.zip › Figure S1.pdf]
